# Supplementary material for: Association between oral microbiome and seven types of cancers in East Asian population: a two-sample Mendelian randomization analysis
Source: Front Mol Biosci. 2023 Nov 21;10:1327893. doi: 10.3389/fmolb.2023.1327893 (PMC10702768; doi:10.3389/fmolb.2023.1327893)
Supplement: Supplementary file 1 [file DataSheet1.ZIP › Supplementary Materials/MR plots of saliva/saliva═╝/Prostate cancer/pheno.437_to_prostate cancer_forest.pdf]

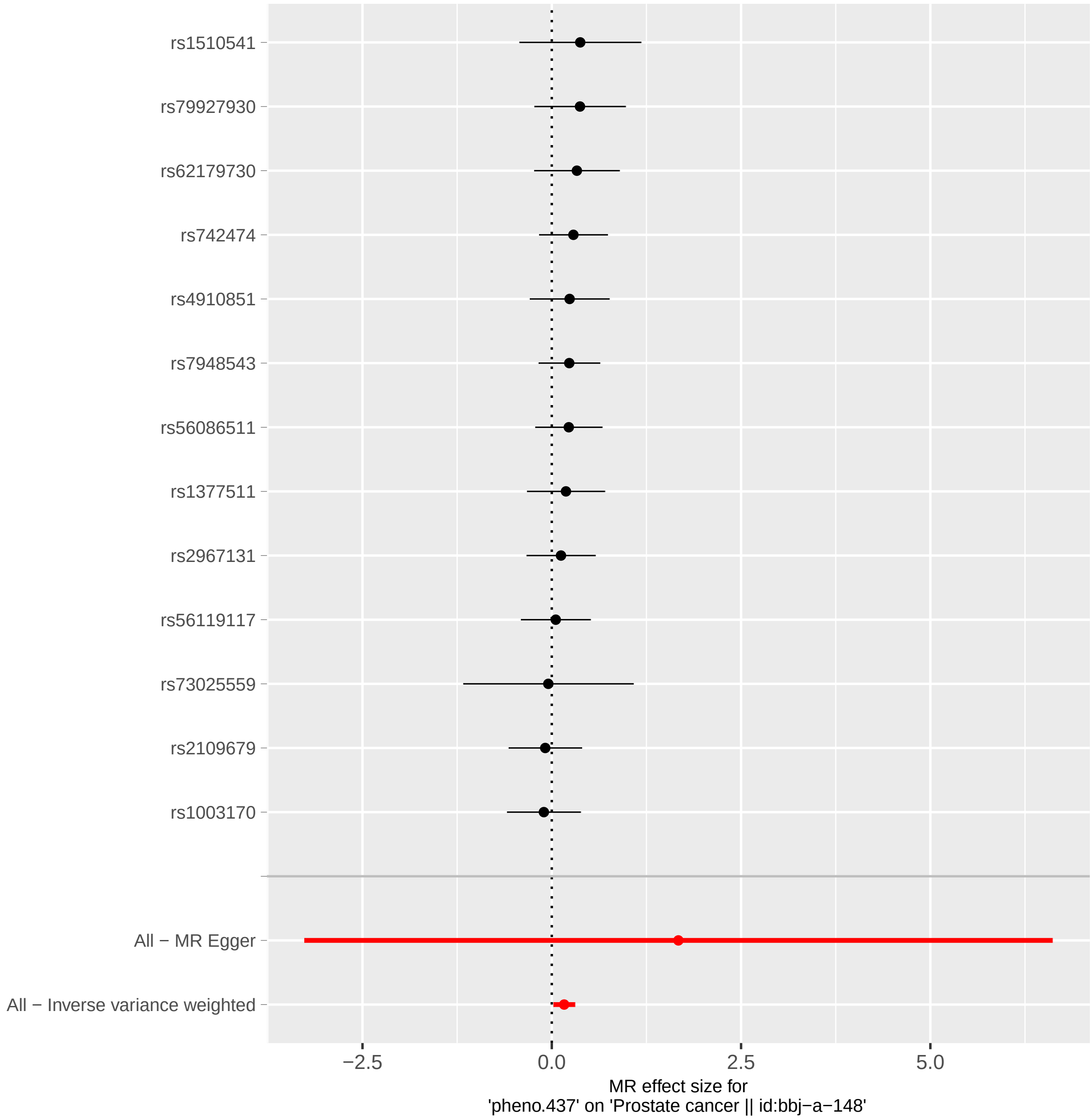

rs1510541
rs79927930
rs62179730
rs742474
rs4910851
rs7948543
rs56086511
rs1377511
rs2967131
rs56119117
rs73025559
rs2109679
rs1003170
All − MR Egger
All − Inverse variance weighted
−2.5
0.0
2.5
5.0
MR effect size for
'pheno.437' on 'Prostate cancer || id:bbj−a−148'
